# A Mixed-Method Case Study to Evaluate Adoption of Clinical Decision Support for Cancer Symptom Management

**DOI:** 10.1055/a-2587-6081

**Published:** 2025-08-22

**Authors:** Jennifer L. Ridgeway, Deirdre R. Pachman, Lila J. Finney Rutten, Joan M. Griffin, Sarah A. Minteer, Jessica D. Austin, Linda L. Chlan, Cindy Tofthagen, Kyle A. Tobin, Veronica Grzegorcyzk, Parvez Rahman, Kathryn J. Ruddy, Andrea L. Cheville

**Affiliations:** 1 Robert D. and Patricia E. Kern Center for the Science of Health Care Delivery, Division of Health Care Delivery Research, Mayo Clinic, Minnesota, United States; 2 Division of Community Internal Medicine, Geriatrics, and Palliative Care, Mayo Clinic, Minnesota, United States; 3 Division of Epidemiology, Department of Quantitative Health Sciences, Mayo Clinic, Minnesota, United States; 4 Department of Physical Medicine and Rehabilitation Research, Mayo Clinic, Minnesota, United States; 5 Division of Epidemiology, Department of Quantitative Health Sciences, Mayo Clinic, Arizona, United States; 6 Division of Nursing Research, Department of Nursing, Mayo Clinic, Minnesota, United States; 7 Division of Nursing Research, Department of Nursing, Mayo Clinic, Florida, United States; 8 Department of Information Technology, Mayo Clinic, Minnesota, United States; 9 Department of Physical Medicine and Rehabilitation, Mayo Clinic, Minnesota, United States; 10 Robert D. and Patricia E. Kern Center for the Science of Health Care Delivery, Mayo Clinic, Minnesota, United States; 11 Division of Medical Oncology, Mayo Clinic, Minnesota, United States

**Keywords:** electronic health records, implementation science, cancer, patient-reported outcome measures, clinical decision support systems

## Abstract

**Background:**

Electronic patient-reported outcome measures (ePROMs) can improve care for people with cancer, but effectiveness hinges on well-supported integration in clinical settings.

**Objectives:**

We evaluated clinician use of specific clinical decision support (CDS) tools in the electronic health record (EHR) designed to facilitate timely, clinically appropriate responses to ePROM scores for six symptoms commonly experienced by cancer patients.

**Methods:**

The parent pragmatic trial, which took place at Mayo Clinic (Rochester, Minnesota, United States) and its affiliated community health care system between March 2019 and January 2023, evaluated the population-level effectiveness and implementation of an ePROM surveillance and EHR-facilitated collaborative care symptom management intervention. The present evaluation used a case study approach with four data sources: (1) clinician interactions with CDS tools abstracted from the EHR; (2) clinician notes identified with an institution-specific textual search tool; (3) qualitative interviews and group discussions with care teams; and (4) administrative records reviewed to identify training and outreach to care teams.

**Results:**

EHR metrics showed very low adoption of CDS tools including alerts and symptom-specific order sets, despite educational outreach and information technology support provided to clinical care teams. Qualitative findings revealed that CDS use was not easy to integrate into busy clinical workflows and highlighted clinician perceptions that the collaborative care intervention provided additional patient support that reduced clinicians' need to utilize CDS tools. They also highlight the importance of contextual factors, including institutional priorities and EHR changes.

**Conclusion:**

This pragmatic clinical trial case study found limited adoption of EHR CDS tools that had been developed to increase clinicians' awareness of and responses to ePROM data. Findings suggest the need to align clinician and organizational implementation strategies, simplify CDS tools to fit practice expectations, and identify and address contextual factors that could undercut strategies like education and peer support. This may be especially important for teams who aim to iteratively evaluate and refine CDS and implementation strategies for multicomponent interventions or introduce new strategies that are responsive to barriers while maintaining scalability.

## Background and Significance

Evidence demonstrates that patient-reported outcome measures (PROMs) and electronic PROMs (ePROMs) may improve clinical outcomes and quality of life among patients with cancer. 1 2 They may also promote patient–clinician communication and patient satisfaction. 3 4 5 6 Unfortunately, research suggests that simply reporting ePROM data to busy clinicians without providing support and resources is a barrier to implementation and limitedly impacts clinical outcomes. 3 7 Reports suggest that patients experience ePROM symptom surveillance as acceptable and beneficial, but respondent burden is a common complaint, particularly when clinicians provide minimal feedback to patients. 8 Coupling ePROM monitoring with collaborative care model (CCM)-based symptom management has proven a more reliable means of reducing symptoms and improving other clinical outcomes. 9 10 The CCM is a validated approach in which a dedicated team assumes transient responsibility for managing a clinical target(s), typically a symptom. 11 Although robustly beneficial, the CCM's high human resources requirements have impeded scaling and dissemination. Incorporating electronic health record (EHR) functionalities for ePROM reporting and clinical decision support (CDS) tools to facilitate core CCM tasks offers potential to lessen resource needs and improve scalability.

The Enhanced, EHR-facilitated Cancer Symptom Control (E2C2) pragmatic trial compared ePROM symptom surveillance paired with an EHR-facilitated version of the CCM to ePROM surveillance alone in reducing six common SPPADE ( S leep disturbance, P ain, P hysical function impairment, A nxiety, D epression, and E nergy deficit [fatigue]) symptoms among cancer survivors. 12 13 The CCM intervention used automated “low-touch” management for moderate symptoms and conventional CCM “high-touch” care for severe symptoms. These management approaches did not require oncology care team participation and were delivered in parallel to oncology care. CDS tools developed for the trial were intended to enable oncology care teams to participate in managing symptoms and partner with CCM teams per their preferences and the extent permitted by their busy practices.

Our CDS development recognized that well-supported integration is critical in clinical settings, 8 particularly with respect to ePROMs given multiple known clinician and practice level barriers to their use. These include system and intervention complexity, time constraints, workflow integration challenges, and limited understanding of how to interpret or apply results in care delivery. 14 15 We considered these issues in developing and testing the E2C2 CDS tools, but our efforts were constrained by a lack of empirical guidance in how to use CDS to advance appropriate clinician responses to ePROM scores. Additionally, there is limited guidance on the best way to combine education, patient engagement, and interactive support strategies to address personal, organizational, and system factors related to implementation success. 15 16 17

## Objective

Our objective was to characterize clinician use of specific EHR CDS tools designed to facilitate timely, clinically appropriate responses to elevated ePROM symptom scores, and thereby guide future development of implementation strategies aimed at overcoming clinician and organizational barriers to EHR CDS adoption.

## Methods

This work was conducted as part of the E2C2 pragmatic clinical trial, which evaluated the effectiveness and implementation of a cancer symptom management intervention based on the CCM. 12 13 This pragmatic trial took place between March 2019 and January 2023 throughout a large multisite health care system that had transitioned from an institution-specific EHR to the Epic EHR between 11 and 21 months prior to practices going live with the intervention. The intervention tested in the E2C2 trial included ePROM surveillance of the six SPPADE symptoms and patient self-management and clinician support when patients reported symptom burden. The ePROMs used an 11-point numeric rating scale and were administered via the EHR to patients with a history of cancer diagnosis, prior to oncology or hematology visits. Automated multimodal symptom-specific self-management materials were provided to patients reporting moderate or worse symptom severity (≥4/10). Patients reporting severe symptoms (≥7/10) were additionally informed, via the reporting system, of the availability of remote, synchronous patient symptom support from a symptom care manager. The intervention condition included CDS tools developed to support the integration of ePROMs into routine clinical practice. Herein, we describe the EHR-enabled CDS and summarize evaluation results of their use. We used a case study approach and mixed methods to examine proposed mechanisms of change, identify conditions that facilitated or hindered CDS adoption, and explore potential mediators and moderators of implementation success. Case studies that explore “how” and “why” questions can uncover contextual factors, such as unique organizational characteristics, that influence change. 18 19

### Clinical Decision Support Tools

Epic EHR CDS developed for the E2C2 trial included tools to: (1) provide oncology clinicians easy access to ePROM scores, (2) describe key contextual information to inform appropriate clinical responses to severe symptom scores, (3) reduce oncology clinician burden in providing evidence-based care for SPPADE symptoms, and (4) enable oncology clinicians to incorporate E2C2 trial self-management and other resources in SPPADE symptom care, including patient referrals to remote symptom care managers. The tools ensured that oncology clinicians were offered multiple means of reviewing SPPADE symptom scores, alerted to the presence of severe symptoms (≥7/10), and provided with resources to coordinate and deliver evidence-based symptom care. 20

#### Presentation of SPPADE Symptoms

**Fig. 1:**
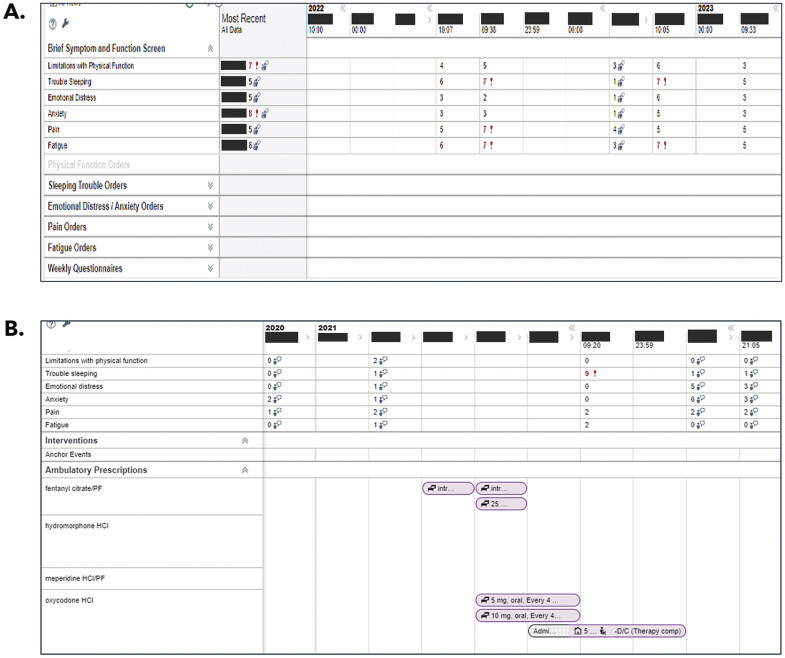
Longitudinal presentation of SPPADE scores in Synopsis view enabled clinicians to identify clinically informative patterns among symptom scores over time. ( **A** ) Drop-down boxes with intervention orders were grouped by symptom and collapsed on opening. ( **B** ) Expanding the drop-down boxes enables users to view stop and start dates for symptom-relevant orders on the same timeline as symptom scores to assess temporal relationships between symptoms and clinical interventions. Epic functionality shown: © 2025 Epic Systems Corporation.

CDS included clinician user interfaces that presented graphic and tabular formats to present longitudinal SPPADE symptom scores. One interface, “Synopsis” in the Epic EHR, was a single place where clinicians could review all the symptom scores and trend them over time if patients had multiple values ( Fig. 1 ). The Synopsis view also presented orders for medications, referrals, procedures, and other interventions with the potential to ameliorate SPADDE symptoms. Orders were grouped in symptom-specific dropdown boxes, which were collapsed on opening the Synopsis view ( Fig. 1A ). Symptom dropdown boxes could be expanded to list all relevant orders placed for a SPPADE symptom ( Fig. 1B ). Order stop and start dates were indicated on the same timeline as symptoms scores, enabling clinicians to evaluate their impact on a patient's symptom. If the symptom care manager was managing a patient's symptoms more frequently, weekly symptom-specific questionnaires would appear at the bottom to display how a patient might be managing.

**Fig. 2:**
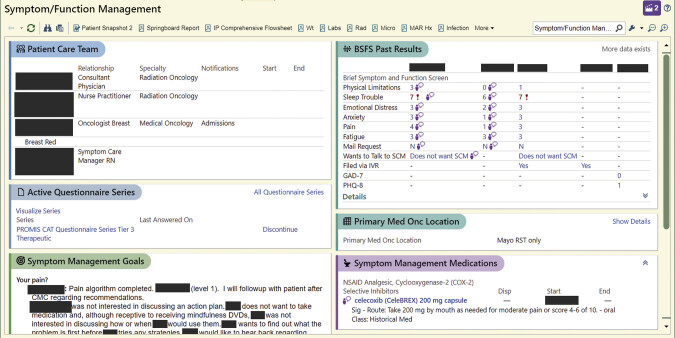
Snapshot summarizes patient ePROM responses over time, as well as actions taken to address symptoms. Epic functionality shown: © 2025 Epic Systems Corporation. ePROM, electronic patient-reported outcome measure.

A second “Snapshot” interface ( Fig. 2 ) was developed as a resource for oncology teams, as well as the symptom care managers, to provide easy access to SPPADE symptom management-relevant clinical information, including symptom scores, and details on patient care team members. Clinicians could also autopopulate their notes with a patient's most recent or last three SPPADE symptom scores using shortcut dotphrases, a function that allows users to type a few characters to automatically expand into a longer phrase. The diversity of note types used by participating medical oncology practices required that each clinician work with their dedicated division or department EHR support staff to add the Smartphrase to their notes. Support was also available from implementation facilitators (referred to in the E2C2 trial as “Symptom Sages”) who were identified from each practice and trained to support the care team from the time they entered the intervention period to study completion. The decision to seek support was left to individual clinicians.

#### Severe Symptom Alerts

**Fig. 3:**
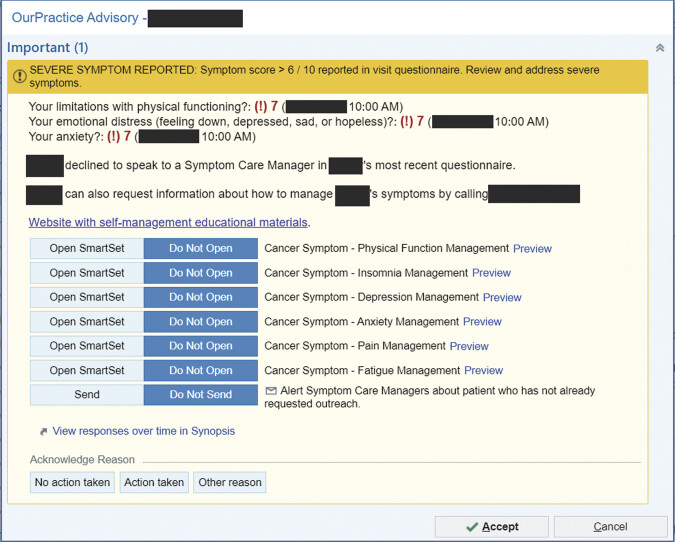
The clinician alert is displayed in the EHR when the patient reports at least one severe SPPADE symptom on an ePROM. It provides links to symptom-specific SmartSets and indicates if a patient requested help from a symptom care manager. Clinicians can acknowledge whether they acted on the alert or not, but no response is required to exit the alert. Epic functionality shown: 2025 Epic Systems Corporation. EHR, electronic health record; ePROM, electronic patient-reported outcome measure.

A clinician-facing alert in the Epic EHR was triggered by report of any severe (≥7/10) SPPADE symptom. The clinician alert displayed, along with sociodemographic and clinical information, on the left-sided vertical navigation pane, “Storyboard” on the EHR view ( Fig. 3 ). The alert indicated severe symptom scores and whether a patient expressed interest in working with a symptom care manager. Additionally, the alert provided links to E2C2 general and symptom-specific resources, e.g., toll-free support line, website, and printable self-management guides. From the alert, clinicians could link to the Synopsis view and order sets for each symptom. They could additionally send an InBasket referral to the E2C2 symptom care managers. The alert was passive, but clinicians were prompted to select “No action taken, Action taken, or Other reason.”

#### Symptom Management Resources

**Fig. 4:**
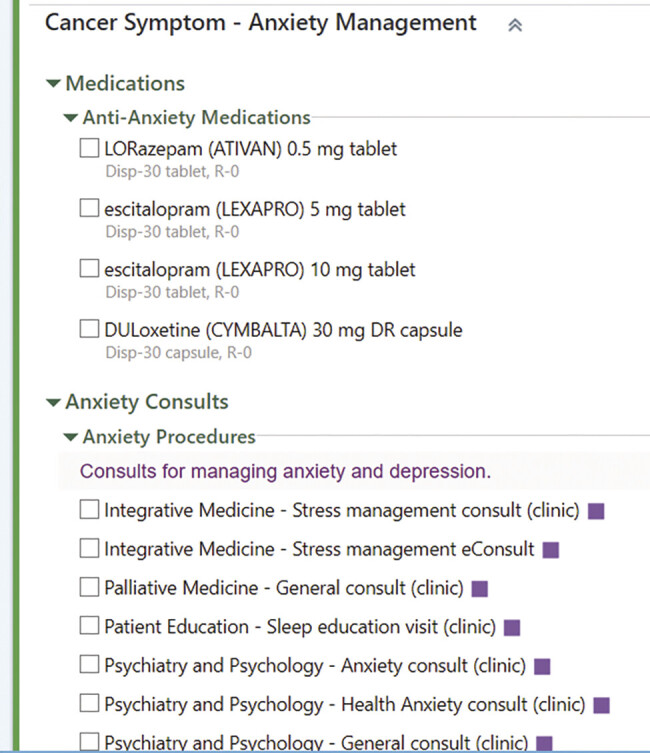
SmartSets provide clinicians with symptom-specific resources and evidence-based treatment and referral options. This example shows the SmartSet for anxiety. Epic functionality shown: 2025 Epic Systems Corporation.

While SPPADE symptom-specific order sets (“SmartSets” in the Epic EHR) were available to clinicians at all times, clinicians were solely prompted by the clinician alert to use them when patients reported severe symptoms. The order sets were informed by current symptom management guidelines and included evidence-based medications, procedures, and specialty referrals, as appropriate, for each symptom ( Fig. 4 ).

### Data Collection and Analysis

We leveraged four data sources for our evaluation. First, data related to frequency and nature of clinicians' interactions with CDS tools were abstracted from the EHR, as shown in Supplementary Table S1 (available in online version only). The Epic Chronicles and Clarity databases were the source of all information related to clinicians' CDS interactions. Information related to alert responses and use of the E2C2 orderset was abstracted from Chronicles. Total number of participating clinicians and relevant notes, as well as clinician site affiliations were determined from Clarity. Second, an institution-specific textual search tool was used to identify notes generated during the trial interval by medical oncology clinicians that included the E2C2 intervention's shortcut dotphrases. The textual search tool has been refined over decades to permit patient-, clinician-, and encounter-specific searches of all content from EHR and non-EHR sources, e.g., clinical, radiology, pathology, sociodemographic, etc. Third, trial administrative records were reviewed to identify the scope of training and educational outreach activities directed at care teams. Fourth, qualitative interviews and group discussions with care teams were captured in audio recordings (transcribed verbatim) and meeting notes. Interview/discussion guides and analysis procedures were guided by the Consolidated Framework for Implementation Research (CFIR), which proposes a set of multilevel factors that may help predict or explain implementation success and inform strategies to address barriers and facilitators. These include factors related to the innovation (e.g., its design, complexity, and adaptability), the setting (e.g., physical/technical/work infrastructure, compatibility with existing workflows or systems, and relative priorities compared with other initiatives), and the people involved (e.g., their capabilities and motivations). 21 Transcripts and notes were imported into qualitative analysis software (NVivo 15, Lumivero, Denver, Colorado, United States) and coded to factors in the framework to facilitate queries.

### Implementation Strategies and Mechanisms of Change

Implementation strategies aimed at clinician use of the E2C2 CDS, as previously reported, 22 are shown in Table 1 along with related outputs and mechanisms of change. Strategies were aimed at improving clinician knowledge and self-efficacy to use ePROM data in patient care, as well as adapting functions (e.g., customizing EHR views in Epic) to make them easier for clinicians to find and use within existing workflows. Strategies were carried out by members of the study team (including clinicians and implementation scientists), Symptom Sages, and EHR support staff affiliated with the oncology practice. 22

**Table 1:** Implementation strategies to increase adoption of clinical decision support components

Implementation strategy (role)	Output	Mechanisms
Train and educate clinicians (study team)	In-person and virtual presentations to care teams with slides showing CDS functions Reference materials including videos demonstrating CDS use in clinical encounters with patients	Care team knowledge of intervention components
Support and remind clinicians (implementation facilitators referred to as “Symptom Sages”)	Kickoff presentations to care teams about intervention components At-the-elbow support by Symptom Sages on use of E2C2 CDS functions Reminders from Symptom Sages about the availability of the interventions in formal and informal settings (e.g., team meetings and workroom or huddle conversations)	Care team knowledge and self-efficacy to use intervention components
Support clinicians and promote compatibility (oncology information technology staff)	At-the-elbow support to help clinicians autopopulate scores in notes and customize activities like Snapshot	Care team self-efficacy Improved access to EHR functions and ease of finding and using them in clinical workflows

Abbreviations: CDS, clinical decision support; E2C2, Enhanced, EHR-facilitated Cancer Symptom; EHR, electronic health record; Control.

Education delivered by the study team included a presentation, given to care teams approximately at the start of their movement into the active implementation period of the cluster-randomized stepped-wedge design, focused on orienting clinicians to the intervention and how to use it. One or more members of the study team continued attending care team meetings quarterly to gather feedback and provide study updates. Symptom Sages were asked to give a kickoff presentation to their care team and to provide regular updates and reminders in formal and informal settings. At-the-elbow support from Symptom Sages and oncology information technology staff aimed to improve self-efficacy and make systems easier to use. In this pragmatic trial, patients and clinicians were included in the intervention period without enrollment and consent procedures as symptom monitoring and management were considered standard of care. There were no targets for CDS use, but clinicians were encouraged to use them as appropriate and the study hypothesized that implementation strategies would result in improvements in implementation outcomes including clinician adoption. 12 13

## Results

The E2C2 trial cohort was comprised of 50,207 patients who received care from a medical oncology clinical during the trial interval from 344 providers. Among this group, 38,644 patients completed one or more ePROMs and 24,874 completed two or more.

### Symptom Characteristics

Roughly 80% (30,315) of the patients who completed at least one ePROM reported a moderate symptom (numeric rating scale score of 4–6/10), and 45% (18,974) of patients reported a severe symptom (numeric rating score ≥7/10) on one or more occasions during the trial period. Only patients in the intervention group had the option to work with a symptom care manager. Of the 12,562 intervention patients who reported severe symptoms, 32% (4020) indicated interest in doing so.

### Use of Clinical Decision Support Components

Metrics demonstrating clinician adoption of the CDS tools are shown in Table 2 . During the trial interval, March 2019 to January 2023, the E2C2 cohort patients had 273,304 completed clinical encounters with 344 providers. All in-person clinical, telecare, and trial-related encounters were included but encounters for refills, chemotherapy, port maintenance, and other needs were excluded. Among these providers, 48 (14%) signed at least one note documenting an outpatient encounter with an E2C2 trial participant that included their recent SPPADE symptom scores autopopulated with a dotphrase. Collectively, the 48 providers who used the dotphrase signed 1401 notes for unique patient encounters, 0.005% of all outpatient encounters.

**Table 2:** Metrics on electronic health record-enabled component use

	*N* (%)
Clinician alert for severe symptoms on ePROM (Our Practice Advisory) a	
Alerts triggered	23,959
Alerts acknowledged	88 (0.367%
Alerts hyperlinked to synopsis	15 (0.063%)
Alerts opened order set	13 (0.054%)
Alerts sent InBasket message	6 (0.025%)
Order set for evidence-based symptom management (SmartSet) a	
Number of orders initiated from E2C2 SmartSets	3
Number of providers placing SmartSet orders	3
Mean E2C2 SmartSet orders placed per provider	1
Shortcut dotphrase (Smartphrases) a	
Number of nonsymptom care manager notes with Smartphrase	1,401 (0.5%)
Number of nonsymptom care manager providers including Smartphrase in notes	48 (14%)
Mean frequency of Smartphrase use by provider	29

Abbreviations: CDS, clinical decision support; E2C2, Enhanced, EHR-facilitated Cancer Symptom Control; EHR, electronic health record; ePROM, electronic patient-reported outcome measure.

a Epic EHR CDS phrasing included in parentheses.

**Fig. 5:**
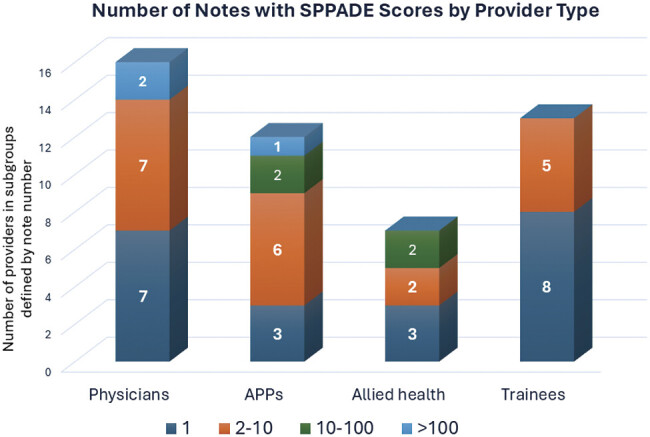
Forty-eight providers signed at least one note with SPPADE symptoms autopopulated. APP, advanced practice provider (e.g., physician assistant or nurse practitioner).

The number of notes per provider, among those who included dotphrases, was highly skewed with a mean of 29, mode of 1, and median of 3. A single provider signed 870 notes. Most signed 1 note ( *n* = 21, 43.8%) or 2 to 10 notes ( *n* = 20, 41.7%) during the trial period. Seven providers signed more than 10 notes. There were two physicians and one advanced practice provider who signed more than 100 notes each, as shown in Fig. 5 .

### Clinical Decision Support Implementation Support

Over the study period, the study team gave almost 100 presentations to care teams. Initially intended to gather feedback and share study updates, quarterly care team discussions shifted (starting in December 2020) to focus on reminding care teams about E2C2 and the available EHR functionality, as well as the role of the symptom care managers. This change was developed in response to reports that clinicians had variable awareness of available CDS tools. Presentations included CDS screenshots and “clinician cheat sheets” summarizing CDS tools and providing instructions for accessing them in Epic. In addition, over the course of the E2C2 trial, the research team increasingly engaged the dedicated EHR support staff responsible for apprising clinicians of EHR updates, answering questions, and customizing their EHR profiles to assist with CDS support and education.

### Qualitative Findings

In addition to quarterly group discussions with care teams, the study team conducted 15 interviews with key informants and members of care teams and 27 group discussions. Qualitative analysis using CFIR domains highlighted personal and work-related implementation challenges, as summarized below, that relate to the primary implementation strategy bundle that targeted clinician knowledge and self-efficacy, as well as access to and ease of using CDS.

#### Clinician Awareness and Relative Priorities

Findings suggest variable awareness of E2C2 CDS components and familiarity with Epic CDS generally. While available, at-the-elbow support was minimally leveraged in the practice setting, and Symptom Sages reported challenges supporting their colleagues with Epic tools, as noted by the following participant: “ *I would personally edit everyone's note to get the dot phrase in there for them, and I think after a while, people just got sick of it and so took it out. I would arrange their Epic to make sure the BPA popped up if there was a high score. But if they're seeing patients and I'm seeing patients, I'm not going to sit there and be like, 'Oh, did you look at their BPA?' I'm not going to hold their hand every step of the way.* ” Likewise, clinicians said they needed to prioritize core tasks during an encounter, as noted by the following care team member, at the expense of E2C2 CDS. “ *There are so many functionalities to [Epic]…And quite frankly, I talk about symptoms, but I think my bigger role is to talk about therapeutics and the bigger picture of their cancer in their lives, and that's really what I focus on.* ” Some clinicians questioned whether their use of Epic functionality was necessary in an effective CCM wherein the remote symptom care manager was supporting patients with severe symptom burden.

#### Workflow Integration and User Design

Clinician participants noted challenges in design and functionality of some EHR components, including difficulty navigating to symptom information, as noted by the following care team member: “ *Snapshot, where you get the numbers, I don't find that helpful…it's more work to get to that point in Epic than it's worth, so I never go there.* ” Clinicians also reported problems fitting CDS use into their workflow, as noted by the following clinician: “ *I think it's just 1) forgetting to do it, and 2) we're already doing a million things in Epic, including the treatment plans and everything like that. I'm talking face-to-face with the patient and pulling up the survey results kind of [gets forgotten].* ” Clinician workflows also varied, as noted in this key informant interview: “ *Some [clinicians] do not open the patient's chart in Epic until after they have seen the patient, while others minimally or never interact with Epic during a patient encounter…In addition, some of the Epic functionality is not user friendly…It is difficult for in-service providers who have little discretionary time, and optimal use of the functionalities often requires practice.* ”

## Discussion

This mixed-method case study revealed very low use of CDS tools enabling clinicians to initiate evidence-based SPPADE symptom management as part of a pragmatic trial intervention seeking to reduce population symptom burden. Although the E2C2 trial demonstrated significant reductions in symptom burden and health care utilization, clinicians' use of CDS does not appear to have mediated these outcomes. Absent a formal mediator analysis, this remains an informal inference, yet the extremely low rates of CDS usage compellingly suggest that this is the case. 23 Our mixed-methods approach identified two overarching insights that can inform future CDS trials of ePROMs and collaborative care.

Recommendation 1: Implementation strategies aimed at clinician education may need to be bundled with organizational EHR strategies and ePROM support to be effective. Implementation strategies were evidence-based and targeted important individual mechanisms of change such as clinician knowledge and self-efficacy. However, there were challenges to engaging clinicians in education and efforts to get input on CDS. Staff turnover, coinciding with the coronavirus disease 2019 (COVID-19) pandemic, led to an increased number of participating clinicians who had not been involved in trial startup and CDS design. Also, trial endorsement was provided by practice leadership, but it was difficult to get broad staff endorsement at all study sites. Inconsistent meeting schedules and the abrupt shift to virtual care that occurred early in the trial with the COVID-19 pandemic limited the conventional modes of obtaining staff input.

Furthermore, certain contextual factors played a bigger role than anticipated and likely contributed to low CDS uptake. First, the clinical practices varied in their exposure to the Epic EHR system, which was newly implemented shortly before the start of this trial. Specifically, the Mayo Clinic sites transitioned from an institution-specific EHR to the Epic EHR 11 months prior to the start of the trial, and the Mayo Clinic health system sites transitioned 17 to 21 months prior to it. Of the E2C2 CDS, only alerts were included in the pre-Epic system, and these were largely confined to potential drug–drug interactions. That meant that providers were potentially less familiar with the Epic EHR, in general, and core CDS concepts employed in the E2C2 trial intervention (e.g., autopopulation of clinical notes) that had not been available in the institution-specific EHR that preceded Epic. Many clinicians were in the process of familiarizing themselves with basic Epic EHR functionalities essential to completing routine workflows when the E2C2 trial intervention went live. The prior institution-specific EHR offered limited CDS functionality, in general, and none related to ePROMs. In fact, the electronification of ePROMs and their viewing within the EHR was generally novel at the institutional level and almost entirely at the specialty practice level. The E2C2 trial's CDS development would have benefited from recognition that the institution's recent abrupt and fairly radical shifts in EHR ePROM and CDS capabilities posed formidable barriers.

Importantly, a better understanding of mechanisms of change, including among bundles of strategies, may be needed to target contextual barriers. 24 CFIR guided analysis of multilevel implementation factors (e.g., personal attitudes related to patient care, impressions of CDS complexity, and organizational structures) that both explain implementation success and guide strategies to address them, but strategies were not bundled based on an understanding of how mechanisms work together. Concomitant leadership support for adoption of ePROMs in care and other organizational strategies to support clinician self-efficacy with the EHR may be necessary to activate educational strategy effects. One challenge that we faced was that institutional IT resources in particular were concentrated on vital capabilities of the new Epic system (e.g., orders, routing) when E2C2 began. New research approaches, such as systems theory, and methods like causal diagramming may help identify key strategies and interdependencies for implementation bundles. 25 26

Recommendation 2: Simple CDS that are well-aligned with clinicians' perceived scope of practice and practice culture may increase buy-in and foster adoption in routine workflows. This study identified technological obstacles (e.g., interfaces that are not user-friendly, inability to customize CDS features) found in other studies of general practitioners and primary care providers, 27 28 as well as concerns about workload, time constraints, or integration into workflow. 28 29 30 Consistent with user-engaged research approaches, clinician leaders on the study team (D.R.P., K.J.R., and A.L.C.) played a key role in CDS development, but they were not representative of all study locations and experiences. Institutional policy and practice culture further presented unanticipated impediments to ePROM-related CDS integration. Specifically, the institution's Epic rollout gave clinicians latitude in assimilating the EHR's data review, documentation, and ordering functions into their workflows. This approach led to marked and poorly characterized variance in how clinicians customized and interacted with the EHR, e.g., some clinicians dictated their notes outside of Epic, had staff enter orders for them, or hovered over clinician practice alerts to see what severe symptom had been reported, rather than clicking on it. Clinicians were able to customize their documentation templates, selection of data presentation interfaces, and patient summary panels (e.g., Epic Storyboard), all of which impacted access to the E2C2 trial CDS. Limited user observation led to false assumptions of greater than actual consistency in Epic use. The efforts of Symptom Sages to support the CDS tools were limited by colleagues' time constraints and low engagement. In addition, the E2C2 grant funding only allowed the study to cover 5% time for each Symptom Sage for three months, and many were unable to utilize this time due to clinical demands, potentially reducing their ability to commit ongoing effort to this role.

Context assessment completed in the preimplementation stage for this study, which was also guided by CFIR, identified anticipated implementation challenges including E2C2 fit in existing practice workflows, 31 even though care team surveys showed high levels of perceived acceptability, feasibility, and appropriateness of E2C2 intervention components overall. 32 The potential for adaptations to CDS tools was limited during the trial, though. Furthermore, bespoke CDS customization is costly, limits potential scale-up, and is often not feasible for institutionally deployed EHR systems. However, limited usability testing and user-centered refinement prior to CDS deployment, in part due to the compressed trial start-up timeline, may be a remediable contributor to poor CDS uptake.

Culture and beliefs regarding oncology clinicians' role in assessing and managing symptoms also varied, even to the extent that some clinicians deferred responsibility for moderate and worse symptoms to the E2C2 symptom care managers. Although the use of ePROMs for oncology symptom surveillance has gained international traction and even emerged as a recommended practice, 8 views on whether and how ePROM data should inform practice continue to evolve. A more thorough characterization of how clinicians viewed their role in ePROM monitoring and symptom management would have tempered our overly optimistic view that ePROM-related CDS would be perceived as offering value. Future research should also consider the potential for patient-facing implementation strategies, including those that engage patients in the design of ePROM systems and other technologies that support symptom management. Future research may explore patients' expectations about how their care team will discuss or respond to ePROMs when completed in the context of a CCM.

These findings may also raise questions about how much clinician adoption of CDS is needed, particularly when the focus of the intervention is to improve symptoms that may not be directly related to the cancer therapy (unlike chemotherapy-related gastrointestinal side effects, for example). This highlights the benefit of a CCM that automates responses to symptoms that often can be underrecognized (SPPADE symptoms) without adding burden to care teams. Further research is needed to determine how clinician adoption of CDS interacts with other interventions components (including those outside the care team, e.g., remote nurse support). Understanding the effect of each component on patient outcomes may also require more investigation of which components are core to intervention effectiveness and which could be adapted to fit local context without reducing the intervention effect.

There are limitations to this work, including challenges to tracking adoption as our primary implementation outcome (i.e., the number of clinicians who take up the practice) 33 34 given our pragmatic approach to intervention delivery and data collection. Limitations in EHR functionality to log intervention component use, for example, meant we were unable to determine how often clinicians read notes or action plans created with the symptom care managers or used SmartSets or Synopsis. We addressed this limitation using a case study approach that leveraged multiple data types and sources to understand uptake of CDS and the role of implementation strategies on their adoption, but future research would benefit from more robust and automated tracking systems.

There are also limitations related to qualitative data collection, which was largely conducted after the start of the COVID-19 pandemic. The predominately virtual nature of data collection (e.g., phone and video-conferencing software) made it difficult to tabulate an accurate count of participants in group discussions, where virtual care team attendance ranged from 2 to 27 (median 12) but attendance changed as people logged onto and off of the meeting and only a subset of care team members actively participated. The number of interviews and group discussions were found to be sufficient for focused analysis to help explain a narrow research question (i.e., CDS adoption), though, in the context of a multiple-method case study. 35

The CDS tools reported here have been maintained since the end of the E2C2 trial. Conversations are ongoing with practice leadership to determine if the CDS will continue to be active (and if so, in what form) in the future, as part of a scaled-back version of the E2C2 intervention to be supported by the Mayo Clinic clinical practice.

## Conclusion

Case study approaches with diverse data sources can identify clinician attitudes, workflows, and behaviors impeding CDS adoption that are not captured in structured EHR data. This case study found limited adoption of EHR CDS tools that had been developed to increase clinicians' awareness of and responses to ePROM data. Findings suggest the need to align clinician and organizational implementation strategies, simplify CDS tools to fit practice expectations, and identify and address contextual factors that could undercut strategies like education and peer support.

## Clinical Relevance Statement

Implementation strategies must be aligned with clinical workflows to improve clinician adoption of CDS tools in multicomponent care models. Clinicians' workflow perspectives and preferences should be ascertained early and often. Ongoing education should be embedded into workflows to address low awareness, while support strategies like peer coaching must be restructured to reduce time burdens on clinicians. Streamlining these processes could enhance the integration of CDS tools into routine care, ensuring more effective symptom management for patients.

## Multiple-Choice Questions

1. Which of the following was a symptom included in the study and the CDS tools?
  - Nausea
  - Sleep disturbance
  - Blurred vision
  - Neuropathy
  **Correct Answer** : The correct answer is option b. This intervention targeted six symptoms of daily living that are common for individuals with a history of cancer, referred to as the SPPADE symptoms ( S leep disturbance, P ain, P hysical function impairment, A nxiety, D epression, and E nergy deficit [fatigue]).
2. What feature was not included in the CDS for this study intervention?
  - Our Practice Advisories triggered when a patient reported a severe symptom
  - Snapshot views of patient symptom reports over time
  - Longitudinal symptom summaries generated by artificial intelligence
  - SmartSets with links to symptom-specific resources, including order sets and referrals
  **Correct Answer** : The correct answer is option c. This intervention included several ways for clinicians to view patient symptom scores and access evidence-based resources, and they were alerted when a patient reported a severe symptom. There were no CDS that leverage artificial intelligence.

**Supplementary Table S1:** Data sources for clinical decision support utilization rates

CDS	Action	Variable	Source
Clinician alert	Opened	Binary (yes/no)	Epic Chronicles
Clinician alert	Acknowledged	Categorical (no action taken, action taken, or other reason)	Epic Chronicles
Clinician alert	Hyperlinked to view symptom scores	Binary (yes/no)	Epic Chronicles
Clinician alert	Opened order set	Binary (yes/no)	Epic Chronicles
Clinician alert	Sent message	Binary (yes/no)	Epic Chronicles
Order set	Symptom management order placed from E2C2 set	Count (clinician number)	Epic Chronicles
Order set	Symptom management order placed from E2C2 set	Count (order number)	Epic Chronicles
Dotphrase	Signed encounter note that included E2C2 dotphrase	Count (note number)	E2C2 Data Explorer
Dotphrase	Signed encounter note that included E2C2 dotphrase	Count (clinician number)	E2C2 Data Explorer

Abbreviations: CDS, clinical decision support; E2C2, Enhanced, EHR-facilitated Cancer Symptom Control; EHR, electronic health record.
