# Supplementary material for: A Mixed-Method Case Study to Evaluate Adoption of Clinical Decision Support for Cancer Symptom Management
Source: Appl Clin Inform. 2025 Aug 22;16(4):804–14. doi: 10.1055/a-2587-6081 (PMC12373467; doi:10.1055/a-2587-6081)
Supplement: Supplementary file 1 — Supplementary Material [file 10-1055-a-2587-6081_26798843.pdf]

Supplementary Table S1 Data sources for clinical decision support utilization rates

CDS	Action	Variable	Source
Clinician alert	Opened	Binary (yes/no)	Epic Chronicles
Clinician alert	Acknowledged	Categorical (no action taken, action taken, or other reason)	Epic Chronicles
Clinician alert	Hyperlinked to view symptom scores	Binary (yes/no)	Epic Chronicles
Clinician alert	Opened order set	Binary (yes/no)	Epic Chronicles
Clinician alert	Sent message	Binary (yes/no)	Epic Chronicles
Order set	Symptom management order placed from E2C2 set	Count (clinician number)	Epic Chronicles
Order set	Symptom management order placed from E2C2 set	Count (order number)	Epic Chronicles
Dotphrase	Signed encounter note that included E2C2 dotphrase	Count (note number)	E2C2 Data Explorer
Dotphrase	Signed encounter note that included E2C2 dotphrase	Count (clinician number)	E2C2 Data Explorer

Abbreviations: CDS, clinical decision support; E2C2, Enhanced, EHR-facilitated Cancer Symptom Control; EHR, electronic health record.

 © 2025. Thieme. All rights reserved.
